# Age-related vulnerability in the neural systems supporting semantic processing

**DOI:** 10.3389/fnagi.2013.00046

**Published:** 2013-09-12

**Authors:** Jonathan E. Peelle, Keerthi Chandrasekaran, John Powers, Edward E. Smith, Murray Grossman

**Affiliations:** ^1^Department of Neurology, University of PennsylvaniaPhiladelphia, PA, USA; ^2^Department of Psychology, Columbia UniversityNew York, NY, USA

**Keywords:** cognitive aging, semantic memory, aging, fMRI, language, compensation

## Abstract

Our ability to form abstract representations of objects in semantic memory is crucial to language and thought. The utility of this information relies both on the representations of sensory-motor feature knowledge stored in long-term memory and the executive processes required to retrieve, manipulate, and evaluate this semantic knowledge in a task-relevant manner. These complementary components of semantic memory can be differentially impacted by aging. We investigated semantic processing in normal aging using functional magnetic resonance imaging (fMRI). Young and older adults were asked to judge whether two printed object names match on a particular feature (for example, whether a tomato and strawberry have the same color). The task thus required both retrieval of relevant visual feature knowledge of object concepts and evaluating this information. Objects were drawn from either natural kinds or manufactured objects, and were queried on either color or shape in a factorial design. Behaviorally, all subjects performed well, but older adults could be divided into those whose performance matched that of young adults (better performers) and those whose performance was worse (poorer performers). All subjects activated several cortical regions while performing this task, including bilateral inferior and lateral temporal cortex and left frontal and prefrontal cortex. Better performing older adults showed increased overall activity in bilateral premotor cortex and left lateral occipital cortex compared to young adults, and increased activity in these brain regions relative to poorer performing older adults who also showed gray matter atrophy in premotor cortex. These findings highlight the contribution of domain-general executive processing brain regions to semantic memory, and illustrate differences in how these regions are recruited in healthy older adults.

Semantic memory refers to our knowledge about the people, places, and objects in our environment, and is a critical component of everyday human behavior. Of particular importance is the fact that semantic memory is multifaceted, encompassing the storage, retrieval, and manipulation of knowledge in a context-relevant manner—that is, involving both content and process (Martin and Chao, 2001; Koenig and Grossman, 2007; Reilly et al., 2011). It is the active and dynamic nature of the semantic memory system that allows us to use our knowledge adaptively in different situations. For example, if asked “Are a strawberry and a tomato the same color?,” color knowledge is required but not necessarily all of the available knowledge about the concept STRAWBERRY (we use capitals to denote a concept) would be accessed. Information pertaining to other aspects of STRAWBERRY-ness—size, shape, taste, preferred climate, and so on—is less relevant in this context, and thus the need to retrieve these details is less necessary. The current study investigates these complementary aspects of content and process as they relate to object knowledge, and how they change during healthy aging.

In the late 19th century, neurologist Carl Wernicke (1885–1886/1977, quoted in Gage and Hickok, 2005) described the link between sensory representation and specific areas of the brain with remarkable prescience:

[T]he memory images of a bell … are deposited in the cortex and located according to the sensory organs. These would then include the acoustic imagery aroused by the sound of the bell, visual imagery established by means of form and color, tactile imagery acquired by cutaneous sensation, and finally, motor imagery gained by exploratory movements of the fingers and eyes.

Recent neuroimaging studies provide compelling supporting evidence that aspects of object knowledge are represented in a distributed manner in the brain (Martin, 2007; Kiefer and Pulvermüller, 2012). That is, regions of visual association cortex appear to be involved in representing visual properties of object concepts (e.g., the color of an apple), regions of auditory association cortex are involved in representing auditory properties (e.g., the sound of thunder) (Bonner and Grossman, 2012), motor association cortex may contribute to the representation of motor properties of action concepts (Grossman et al., 2008), and so on. We hypothesize that these modality-specific representations are then integrated through heteromodal regions of association cortex that act as conceptual hubs where feature knowledge from multiple modalities can be integrated to support the representation of an object concept (Patterson et al., 2007; Binder and Desai, 2011; Bonner et al., 2013). Although these sensory-motor representations can be complemented by amodal, grammatical, and other types of information (Postle et al., 2008; Bedny and Caramazza, 2011; de Zubicaray et al., in press), we believe they play an important role in the richness of concept representation. Thus, there is converging evidence from functional magnetic resonance imaging (fMRI) studies of healthy adults and structural imaging studies of patients with focal neurodegenerative conditions that the content of semantic memory is supported in a distributed fashion linked in part to modality-specific association cortices, based on our acquired sensory-motor experience and knowledge about object properties.

However, sensory information is not the only guiding force in semantic memory. The idea that conceptual knowledge relies not only on perceptual features has a long history in both philosophy and neuroscience. In Plato's dialogue *Phaedo* (c.360 BCE), Socrates muses about the unreliability of sensory information, concluding that “thought” plays a key role:

What again shall we say of the actual acquirement of knowledge? Is the body, if invited to share in the inquiry, a hinderer or a helper? I mean to say, have sight and hearing any truth in them? Are they not, as the poets are always telling us, inaccurate witnesses?… must not existence be revealed to her in thought, if at all?

Contemporary neuroscience affirms the critical role of retrieval and evaluation in semantic memory (Martin and Chao, 2001; Koenig and Grossman, 2007; Jefferies et al., 2008). In contrast to the modality specificity exhibited by sensory-motor information, these executive aspects of semantic memory are supported to a large extent by regions of frontal cortex (Devlin et al., 2003), though are likely to include other regions of temporal and parietal cortex as well (Whitney et al., 2011a,b). Depending on the particular task, frontal activity may be characterized as selecting from among competing alternatives (Thompson-Schill et al., 1997), identifying the criteria necessary for membership in a semantic category (Koenig et al., 2005; Peelle et al., 2009) or strategic decision-making that incorporates probabilistic knowledge (McMillan et al., 2012). While these frontal regions may not be involved directly in semantic representation, they appear to contribute to active processes such as retrieving and interacting with object representations in a task-relevant manner. Such active processing is critical to our everyday use of semantic knowledge.

How might these complementary pieces of semantic memory be affected as we age? Normal aging is associated with decreases in both gray matter volume (Good et al., 2001; Raz et al., 2005; Fjell et al., 2009) and white matter integrity (Salat et al., 2005; Madden et al., 2009). The changing structure of the brain is paralleled by age-related changes in cognitive function, including domains of working memory, processing speed, inhibitory control, and other executive processes thought to be mediated by frontal lobe functioning (Grady, 2008; Reuter-Lorenz and Park, 2010). Moreover, fMRI patterns of brain activation during task performance by older adults frequently differ from those seen during performance of the same task by young adults. One pattern often noted in older adults is bilateral activation in brain networks that are ordinarily unilateral in young adults (Cabeza, 2002), a finding that also holds for sentence processing in healthy adults (Wingfield and Grossman, 2006; Peelle et al., 2010; Tyler et al., 2010). One promising explanation for these findings is that bilateral activation in older adults may be related in part to a compensatory process that allows older adults to maintain high levels of behavioral performance by recruiting additional brain tissue in the face of age-related neuroanatomic atrophy. However, age-related structural brain changes are widespread, and show significant individual variability (Peelle et al., 2012). Furthermore, normal aging is ubiquitously associated with increased behavioral variability on numerous tasks (Hultsch et al., 2002; MacDonald et al., 2003), suggesting differing cognitive ability linked to neuroanatomical change (Hedden and Gabrieli, 2004).

Against this background, it appears that semantic knowledge is relatively preserved in older age (Ackerman and Rolfhus, 1999). It is possible that older adults maintain reliable processing of semantic information through the use of brain networks that extend beyond those seen in young adults. Previous research on the neurobiology of semantic memory and aging has provided some intriguing suggestions.

An fMRI study by St-Laurent et al. (2011) directly compared young and older adults' neural activity while performing several different types of memory tasks. The semantic task was a general memory retrieval task in which subjects were shown a photograph and asked a general knowledge question related to the theme of the picture. Although the authors found age-related differences in episodic and autobiographical memory tasks, none were present in the semantic memory task. Similarly, Maguire and Frith (2003) found comparable patterns of brain activity in young and older adults when accessing general knowledge, despite age-related differences in autobiographical memory. However, these tasks tested retrieval of general knowledge, rather than evaluating features reflecting object knowledge. The latter may require different underlying processes that rely more on regions of frontal cortex.

In a more specific test of active semantic evaluation, Stebbins et al. (2002) conducted an fMRI study in which they presented young and older adults with two word judgment tasks: In a semantic task, subjects decided whether words were abstract or concrete in meaning, whereas in a non-semantic task they decided whether the words were written in uppercase or lowercase letters. The authors found increased bilateral frontal activity for the semantic task, consistent with involvement of frontal regions in accessing and evaluating semantic knowledge. They also reported suggestive results of differing extents of activity, with young adults showing a larger extent of activity in the left hemisphere than older adults. In another study reporting age differences in semantic processing, Kounios et al. (2003) presented single words naming an animal or a manufactured object that were judged in a semantically neutral manner by young and older adults. They found greater activation in older adults compared to young adults in the cingulate for manufactured objects. Thus, although the existing imaging literature is mixed regarding the degree to which aging affects the brain systems supporting semantic memory, it suggests age-related changes in semantic processing.

In a previous fMRI experiment we investigated the large-scale brain networks associated with accessing object knowledge in healthy young adults (Grossman et al., 2013). In the current study, we extend these findings by investigating the degree to which normal aging impacts the brain regions involved in accessing object knowledge. We presented young and older adults with pairs of written words and an attribute for which the concepts were evaluated. For example, given the pair “strawberry—tomato” and the attribute “color,” participants would be expected to respond “same” since both objects are red. However, given the same stimulus object pair and the attribute “shape,” participants would be expected to answer “different” because a strawberry is somewhat pointed whereas a tomato is spherical. Stimulus objects differed categorically, being drawn from either manufactured objects or natural kinds, and could be evaluated on either color or shape attributes. We tested the degree to which semantic processing differed as a function of object identity (categorical information), attribute (shape vs. color), and individual subject characteristics (age and performance level).

## Materials and methods

### Subjects

We scanned 18 young adult subjects, ranging from age 18–33 years (mean = 24.4; 9 males), and 21 older adult subjects between the ages of 50–78 years (mean = 65.0; 9 males). The young adults were previously reported in Grossman et al. (2013). Older adults were recruited from the community using flyers and presentations at community centers. Written informed consent was obtained from all subjects according to a protocol approved by the University of Pennsylvania Institutional Review Board (protocol 808437). Cognitive performance was assessed in older adult subjects using the Mini-Mental State Examination (MMSE, max = 30, group mean = 28.9, *SD* = 1.3, *n* = 19). Subsets of older adult participants additionally completed language-related tests as part of a neuropsychological battery: the FAS test of verbal fluency and a semantically-guided category naming fluency task. *Z*-scores were generated based on a previous cohort of 45 demographically-comparable healthy older adults (23 females) who were screened to avoid any history of head injury, vision or hearing loss, seizure, hyper/hypothyroidism, heart disease, lung disease, kidney disease, stroke/TIA, cancer, known toxin exposure, recreational drug use, and depression, and had MMSE scores of 28 or better (mean = 29.32). They were matched (*p* > 0.6) for sex, age, and education with the older adults used in this study, who demonstrated normal performance (FAS *z*-score: mean = −0.2, *SD* = 1.2, *p* = 0.53, *n* = 14; category naming fluency *z-score:* mean = −0.3, *SD* = 1.2, *p* = 0.33, *n* = 13). We were unable to collect neuropsychological data in all subjects in the current study for a variety of reasons (e.g., limited time availability, technical limitations). All subjects were right-handed native English speakers, had normal (or corrected-to-normal) vision, and had good general health and no history of neurological difficulty, as established by a pre-scan self-report screening form.

### Materials

We created pairs of printed nouns, half of which denoted natural kinds and half manufactured artifacts. Natural kinds consisted of fruits, vegetables and animals, and manufactured artifacts consisted of implements, sports equipment and means of transportation. In a preliminary assessment, nine subjects who did not participate in the main study evaluated a large number of word pairs for similarity of color and shape using a 7-point similarity scale. From these items, we selected 160 pairs with the most consistent judgments across subjects. Stimuli were evenly distributed across natural kinds and manufactured artifacts. Frequency scores and familiarity ratings obtained from a group of 20 young adults who did not participate in the main study were used to match lists of items, and no significant differences were found between the lists for each subcategory, or between the lists for natural kinds and manufactured artifacts, or between the lists for the conditions of shape and color.

### Procedure

**Figure 1:**
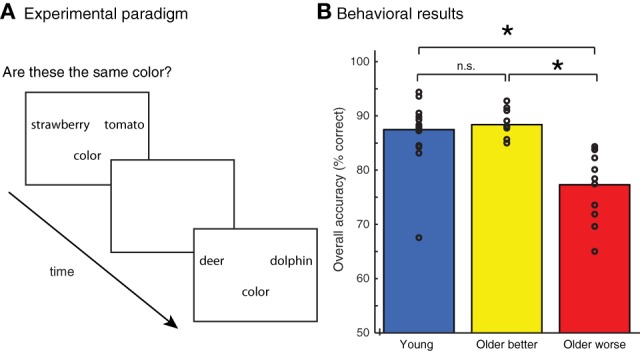
**(A)** Experimental paradigm. Pairs of words were presented and participants were asked whether they matched on an object attribute (color or shape). **(B)** Accuracy of judgments on all tasks for young adults and two groups of older adults, divided into better and poorer performers based on median-split performance. Individual subject scores are shown as unfilled circles. ^*^ indicates a difference between groups at *p* < 0.001.

The experimental procedure is shown schematically in Figure 1A. Each trial began with a 500 ms crosshair followed by presentation of a pair of stimuli. Stimuli remained on the screen for 2.5 s or until subjects responded using a keypad to indicate “same” or “different.” Between each trial, there was an interval of 0, 3, 6, 9, or 12 s, during which time a blank, white screen was displayed in order to produce variability in the timing of the hemodynamic response. Subjects were trained in advance on the experimental method with several practice items, and all subjects appeared to understand the task and the procedure for indicating their judgments.

Presentation was blocked by task in order to minimize executive control demands associated with switching between materials or between probes. The first run involved the word-judgment task, which asked subjects to compare pairs of written object nouns based on a perceptual attribute. Runs began with a question for 3 s indicating the attribute to be compared during the block (e.g., “Are these the same color?” or “Are these the same shape?”), and the relevant property (i.e., “color”/”shape”) was written below each word pair during presentation of the remainder of the stimuli for a run. An event-related design was used, and 80 word pairs (40 pairs of natural kinds, 40 pairs of manufactured objects) were presented in a fixed pseudorandom order for each run.

Following the word-judgment runs, subjects performed two low-level control tasks, a visual form judgment task and a pseudoword comparison task. As our focus is on group differences during the semantic processing task, we focus here on brain activation during the word evaluation tasks.

### MRI data acquisition and analysis

MRI data were acquired on a Siemens Trio scanner (Siemens Medical Systems, Erlangen, Germany) at 3T, beginning with acquisition of a T1-weighted structural volume using a MPRAGE sequence (repetition time [*TR*] = 1620 ms, echo time [*TE*] = 3 ms, flip angle = 15°, 1 mm slice thickness, 192 × 256 matrix, voxel size = 0.98 × 0.98 × 1 mm). Blood oxygenation level-dependent functional MRI images were acquired with 3 mm isotropic voxels, flip angle = 15°, *TR* = 3 s, *TE*_eff_ = 30 ms, and a 64 × 64 matrix.

We analyzed the fMRI data using SPM8 (Wellcome Trust Centre for Neuroimaging, London, UK). For each subject, images were realigned to the first image, coregistered to the structural image, and normalized to Montreal Neurological Institute (MNI) space using unified segmentation (Ashburner and Friston, 2005), including resampling to 2 × 2 × 2 mm voxels, and spatially smoothed with a 9 mm full-width at half maximum (FWHM) Gaussian kernel. Responses to events were modeled using a canonical hemodynamic response function, and movement parameters were included as covariates of no interest. In the current analyses we consider only activity related to correct responses (incorrect responses were modeled using a separate condition). Parameter estimates from single-subject analyses were brought to second-level random effects analyses for making group inferences. Unless otherwise specified, these were thresholded at a voxelwise threshold of *p* < 0.001 (uncorrected), FDR corrected for multiple comparisons at the cluster level (*q* < 0.05) using random field theory (Worsley et al., 1992; Friston et al., 1994; Chumbley and Friston, 2009). For effects across all subjects, groups were weighted equally (e.g., contrast weight of [1/3 1/3 1/3] when looking at 3 groups). Statistical maps for the MRI analyses were rendered on 3D MNI-space templates from SPM8. For results tables, X (left-right), Y (posterior-anterior), and Z (inferior-superior) coordinates refer to locations in MNI stereotactic space.

For the structural MRI analysis we segmented each subject's T1-weighted image into 5 tissue types based on tissue intensities and tissue probability maps, as implemented in SPM8's “new segment” function using a unified segmentation approach (Ashburner and Friston, 2005). Default values were used for segmentation, except that the data were sampled every 1 mm (instead of the default 3 mm) and moderate Markov random field cleanup (value of 2) was used. Subject's brains were normalized using DARTEL (Ashburner, 2007) with total gray matter preserved during spatial normalization to MNI space (i.e., modulated gray matter images). The normalized images were smoothed at 9 mm FWHM. In region of interest (ROI) analyses we first factored out total intracranial volume (TIV) from the data prior to performing *t*-tests, and we included an approximation of TIV (summed gray matter, white matter, and CSF volumes) as a covariate of no-interest in whole brain analyses (Barnes et al., 2010). No global gray matter covariates were included in the analysis, as we were interested in absolute amount of gray matter difference (Peelle et al., 2012).

## Results

### Behavioral results

Subjects' accuracy on the behavioral task is shown in Figure 1B. Although accuracy was generally high, young adults performed significantly better than older adults, *t*_(37)_ = 2.24, *p* < 0.05. However, there was a significant amount of variability within the group of older adults. We performed a median split on the older adults, dividing them into two groups based on their overall behavioral accuracy: older adults performing above the median were classified as “better performing” older adults, those below the median “worse performing” older adults. We found that the better-performing older adults (*n* = 10) performed equivalently to the young adults, *t*_(26)_ = 0.48, n.s. However, the accuracy for the worse-performing older adults (*n* = 11) was significantly poorer than young adults, *t*_(27)_ = 4.4, *p* < 0.001. The accuracy of the worse-performing older adults was also significantly poorer than that of the better-performing older adults, *t*_(19)_ = 5.0, *p* < 0.001. There was no difference in age between the better performing group (mean = 63.5 years) and worse performing group (mean = 66.4 years), *t*_(19)_ = −0.80, n.s.

### fMRI results

**Figure 2:**
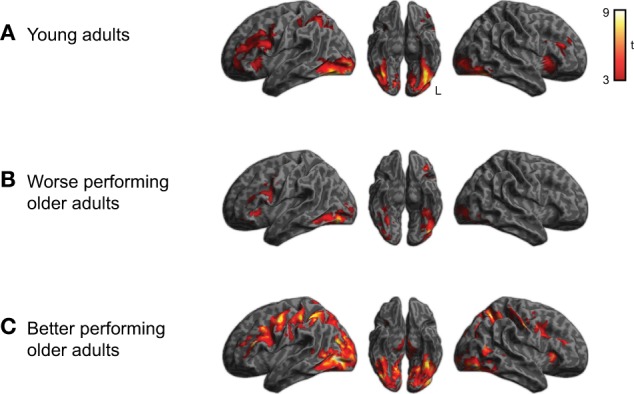
**Activation for performing semantic judgments (collapsed across category) relative to rest for each of the three subject groups (voxelwise *p* < 0.001, cluster-level corrected *q* < 0.05). (A)** Activity for young adults. **(B)** Activity for worse-performing older adults. **(C)** Activity for better-performing older adults. Older adults were divided into groups based on median split performance of the behavioral data.

**Figure 3:**
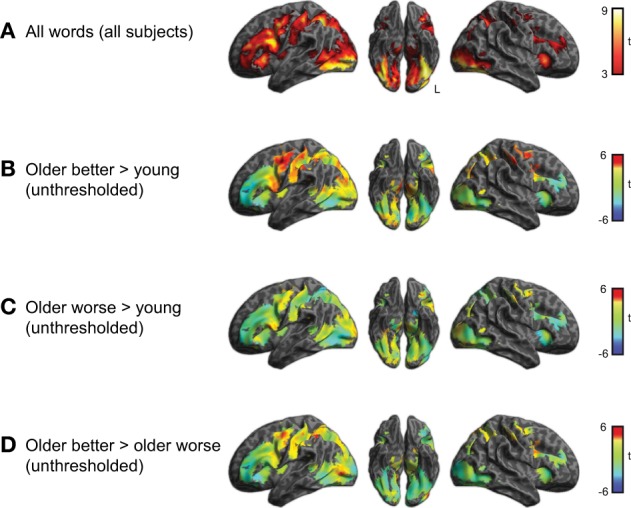
**(A)** Brain activity associated with semantic task for young and older adults together (voxelwise *p* < 0.001, cluster-level corrected *q* < 0.05). This result was used to mask the unthresholded comparisons shown in the remaining panels. **(B)** Comparison between better-performing older adults and young adults. Positive *t*-values indicate more activity in the group of better performing older adults than young adults, negative values more activity in young adults than older better performing adults (none of which was significant). **(C)** Comparison between worse-performing older adults and young adults. **(D)** Direct comparison between better and worse performing older adults.

We first examined the activity associated with semantic processing for all subjects across all word categories, shown in Figure 2 (for each group of participants) and Figure 3A (averaged over participant groups; see also Table 1). Consistent with earlier work, semantic processing resulted in increased activity across a number of brain regions, including inferior temporal cortex and left dorsolateral prefrontal cortex (cluster-level *q* < 0.05), as we found in our previous study (Grossman et al., 2013).

**Table 1:** **Maxima for fMRI activity related to semantic processing task for all subjects**.

**Cluster size (μl)**	**Region**	**Peak coordinate**			***Z*-score**
		***X***	***Y***	***Z***	
162088	Left fusiform gyrus	−42	−70	−14	7.52
	Left inferior parietal cortex	−28	−52	44	7.51
	Left fusiform gyrus	−36	−82	−14	7.48
	Left fusiform gyrus	−40	−62	−14	7.46
	Left fusiform gyrus	−40	−78	−16	7.40
	Right inferior occipital cortex	38	−78	−12	7.38
	Left occipital cortex	−28	−96	−4	7.30
	Left inferior occipital cortex	−48	−80	−6	7.27
	Left inferior occipital cortex	−44	−82	−6	7.22
	Right fusiform gyrus	38	−56	−22	7.08
	Left precentral gyrus	−44	0	38	7.06
	Right fusiform gyrus	34	−60	−20	7.03
	Right occipital cortex	26	−96	−2	7.00
	Left fusiform gyrus	−32	−50	−24	6.98
	Left inferior frontal gyrus	−44	8	30	6.97
	Left cerebellum	−26	−70	−20	6.97
33480	Left precentral gyrus	−44	−0	−38	7.06
	Left inferior frontal gyrus	−44	8	30	6.97
	Left inferior frontal gyrus	−40	12	24	6.81
	Left inferior frontal gyrus	−46	10	22	6.78
	Left inferior frontal gyrus	−42	28	16	6.59
	Left insula	−30	20	2	6.44
	Left inferior frontal gyrus	−34	30	−10	6.28
	Left inferior frontal gyrus	−48	46	2	5.15
2718	Right insula	32	22	0	6.25
2328	Right frontal operculum	40	2	28	5.99
	Right frontal operculum	56	18	34	4.75
5736	Right thalamus	16	−2	6	5.87
	Right thalamus	14	−14	8	5.84
	Right thalamus	12	−10	6	5.83
	Right thalamus	20	−10	14	5.43
3544	Right angular gyrus	32	−56	46	5.82
	Right occipital cortex	32	−68	28	5.16
4496	Supplemental motor area	−2	16	48	5.70
	Supplemental motor area	−6	6	54	5.38
	Anterior cingulate gyrus	8	22	38	4.95
1120	Right middle frontal gyrus	46	42	24	5.48
304	Right middle frontal gyrus	36	−2	64	5.27
96	Left middle temporal gyrus	−52	−42	8	4.67

Clusters from thresholding at voxelwise *p* < *0.05*, FWE corrected. Here and elsewhere, X, Y, and Z refer to coordinates in MNI space.

Within the region identified in Figure 3A, we then compared activity for the subgroups of subjects. This comparison is shown in Figure 3 and listed in Table 2. The better performing older adults showed significantly (*q* < 0.05 whole-brain cluster-corrected) increased activity relative to young adults in bilateral premotor cortex (Figure 3B), which was not evident in the worse-performing older adults (Figure 3C). A direct comparison of the two groups of older adults, shown in Figure 3D, found a trend toward greater activity in the better performing older adults in premotor, inferior parietal, and lateral occipital cortex, reaching *p* < 0.001 uncorrected (see Table 2).

**Table 2:** **Maxima for fMRI activity comparing better performing older adults with other groups**.

**Cluster size (μl)**	**Region**	**Peak coordinate**			***Z*-score**
		***X***	***Y***	***Z***	
BETTER PERFORMING OLDER ADULTS > YOUNG ADULTS^*^					
9000	Left precentral gyrus	−46	−6	42	5.60
	Left precentral gyrus	−30	−10	64	3.80
	Left precentral gyrus	−32	−28	66	3.80
4624	Right postcentral gyrus	60	−18	46	4.54
	Right inferior parietal cortex	52	−28	52	3.96
	Right supramarginal gyrus	60	−20	30	3.39
6448	Cingulate gyrus	10	−18	40	4.41
	Cingulate gyrus	−8	−20	44	4.05
	Supplemental motor area	−6	−4	54	3.96
10728	Right precentral gyrus	52	4	40	4.30
	Right precentral gyrus	38	4	34	4.10
	Right superior frontal gyrus	24	−2	52	3.92
3256	Left inferior parietal cortex	−52	−34	44	4.00
	Left inferior parietal cortex	−48	−46	48	3.84
	Left supramarginal gyrus	−54	−30	36	3.79
BETTER PERFORMING OLDER ADULTS > WORSE PERFORMING OLDER ADULTS^**^					
152	Left inferior occipital gyrus	−48	−80	−4	3.93
688	Left inferior parietal cortex	−48	−46	48	3.80
304	Left precentral gyrus	−44	−6	50	3.63
216	Right precentral gyrus	42	6	30	3.38

* Clusters from thresholding at voxelwise p < 0.001, whole–brain cluster level corrected q < 0.05.

** Voxelwise *p* < 0.001 (uncorrected).

**Figure 4:**
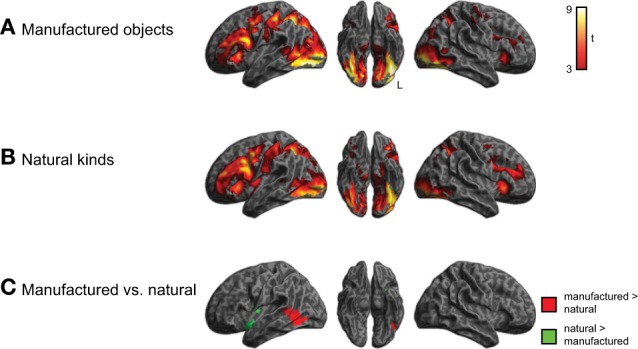
**Brain activity for judgments about manufactured objects and natural kinds, across all three groups of subjects (voxelwise *p* < 0.001, cluster-level corrected *q* < 0.05). (A)** Activity for manufactured objects. **(B)** Activity for natural kinds. **(C)** Comparison of activity for manufactured objects and natural kinds. The category × group interaction was not significant.

We next conducted a comparison of categorical semantic processing, examining activity for manufactured objects and natural kinds separately, shown in Figure 4 and listed in Supplemental Tables 1, 2. We identified category-specific differences in semantic processing in which all subjects showed more activity (*q* < 0.05 whole-brain cluster-corrected) in left lateral temporal cortex (middle and inferior temporal gyri) for manufactured objects compared to natural kinds. Conversely, we observed greater activity (*q* < 0.05 whole-brain cluster-corrected) for natural kinds in left insula. There was not a significant category × group interaction.

**Figure 5:**
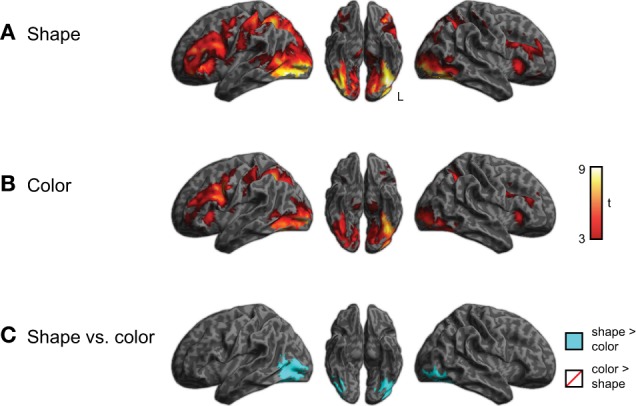
**Brain activity for judgments regarding shape or color, across all three groups of subjects (voxelwise *p* < 0.001, cluster-level corrected *q* < 0.05). (A)** Activity for shape judgments. **(B)** Activity for color judgments. **(C)** Comparison of activity for shape and color judgments. The task × group interaction was not significant.

In addition to processing that differed by category, we also investigated whether semantic processing might differ as a function of the queried property (i.e., shape vs. color). To address this we compared activity for the shape and color task, collapsing across semantic category, as shown in Figure 5 and listed in Supplemental Tables 3, 4. Although shape and color processing resulted in similar patterns of overall activity, we found significantly more activity (*q* < 0.05 whole-brain cluster-corrected) for the shape task in ventral and lateral visual regions (light blue). No regions showed increased activity for color > shape judgments, nor was there a task × group interaction.

### Structural MRI results

**Figure 6:**
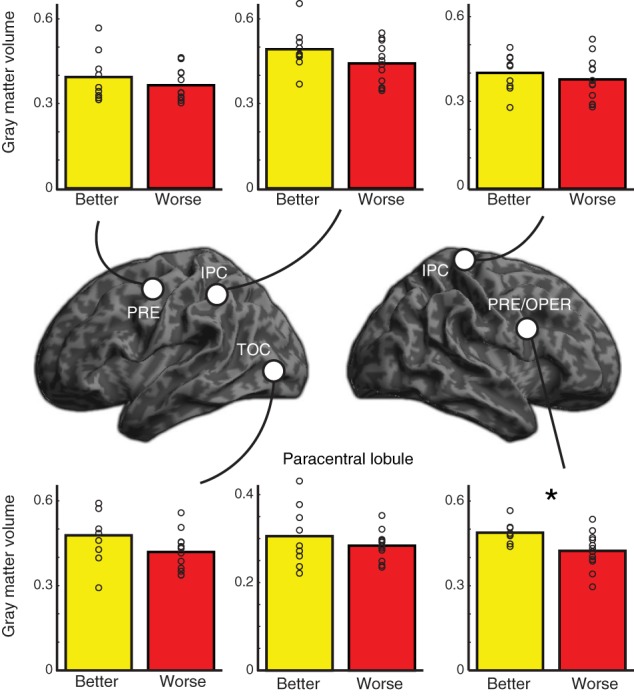
**Location of regions of interest (ROIs) used in gray matter analysis along with extracted gray matter data for better performing and worse performing older adults**. *P*-values are one one-tailed *t*-tests for better-performing older adults having more gray matter than worse-performing older adults; ^*^ indicates significant after controlling for multiple comparisons (see Table 3). Individual subjects' extracted gray matter for each region are shown as unfilled circles. PRE, precentral gyrus; IPC, inferior parietal cortex; TOC, temporal occipital cortex; OPER, operculum. The paracentral lobule ROI is not shown on the renderings.

Given the increased activity in better performing older adults, we conducted two complementary analyses to see whether there were differences in regional gray matter between the two groups of older adults. We first conducted a focused ROI analysis in which we extracted gray matter volume for each of the 6 clusters that showed increased activity for better performing older adults compared to worse performing older adults at a voxelwise threshold of *p* < 0.001 (uncorrected and unmasked) (similar to Figure 3D, but not restricted to regions showing an overall effect). We conducted independent samples one-tailed *t*-tests for each region on gray matter residuals, having factored out effects of TIV, Bonferroni corrected for multiple comparisons across the 6 regions and listed in Table 3. The ROIs used and raw extracted gray matter is shown in Figure 6. Results in most regions were in the predicted direction (with better-performing older adults showing more gray matter), a finding that was significant in right precentral gyrus, *t*_(19)_ = 3.05, uncorrected *p* = 0.003.

**Table 3:** **Increased gray matter volume in better performing older adults relative to worse performing older adults**.

**Region**	**ROI peak coordinate**			***t*-statistic**	***P*-value**
	***X***	***Y***	***Z***		
Left temporal occipital cortex	−48	−80	−4	1.67	0.056
Left inferior parietal cortex	−48	−46	48	1.57	0.067
Left precentral gyrus	−44	−6	50	0.81	0.215
Paracentral lobule	0	−28	70	0.84	0.205
Right inferior parietal cortex	50	−42	56	0.55	0.294
Right precentral gyrus/operculum	42	6	30	3.05	0.003^*^

* Significant after Bonferroni correction.

To ensure we were not missing differences elsewhere in the brain, we also conducted a whole-brain analysis comparing gray matter volume between the two groups. We used a permutation test (Nichols and Holmes, 2001) with 10,000 iterations as implemented in FSL 5.0.2.2's *randomize* function, using threshold-free cluster enhancement to identify significant differences between groups (Smith and Nichols, 2009), including TIV as a covariate. This analysis did not yield any significant results.

## Discussion

In the current study we investigated age-related changes in neural support for semantic processing using a language-based task that taps object knowledge. Our findings are consistent with a large-scale network encompassing visual areas important for the representation of sensory features associated with object knowledge (in inferior and lateral temporal and occipital cortex), and executive regions that contribute to semantic processing (centered in bilateral premotor cortex and left DLPFC). These regions are anatomically linked by a dorsal pathway through the superior longitudinal and/or arcuate fasciculi, and a ventral pathway through the inferior frontal-occipital fasciculus (de Schotten et al., 2012; Grossman et al., 2013). We also found that the brain regions supporting the content and processing of object knowledge interact with the individual abilities of older adults performing the task. Thus, we observed increased frontal activation in older adults with good semantic performance relative to young adults. However, there was reduced activation in prefrontal regions in older adults with poorer semantic performance, corresponding to frontal atrophy in this subgroup of older adults, and we also found reduced temporal-occipital activation in this subgroup of older adults. Although our results must be interpreted within the context of the modest size of our participant groups, we argue below that our findings support the hypothesis that some older adults show increased activation in prefrontal brain regions in order to support strategic cognitive processes that help maintain performance on measures such as semantic judgments (Grady, 2008; Reuter-Lorenz and Park, 2010).

### Processing in sensory-perceptual regions during processing of object knowledge

With respect to semantic content, there is an emerging consensus that object concepts rely at least in part on modality-specific representations of feature knowledge about these objects (Martin, 2007; Bonner and Grossman, 2012; Kiefer and Pulvermüller, 2012; Rodriguez et al., 2012). Of greatest relevance here are temporal and occipital regions in ventral-lateral visual association cortex that are important for both perceptual processing of shape and color (Malach et al., 1995; Zeki and Marini, 1998) and the representation of color and shape features of object concepts (Chao et al., 1999; Simmons et al., 2007; Grossman et al., 2013). In the present study, we found activation of temporal-occipital cortex during judgments of color and shape features of printed object names. We used word stimuli to minimize activation associated with visual perception of stimulus pictures. Although we believe that activation in this region likely reflects accessing visual knowledge involved in concept representation, we cannot rule out the possibility that these activations reflect in part the generation of a mental visual image of the named objects (even so, this would entail a semantically-mediated visual process). Ventral-lateral temporal-occipital cortex recruitment was present in both young and older participants as part of a common pattern of semantic processing. Nevertheless, the subset of older adults with better performance appeared to activate a portion of this area more than poorer-performing older adults. Thus, increased occipital activation in better performing older adults contributed in part to their ability to equal performance of young adults, while poor performing older adults were unable to equal the performance of young adults without increased activation in this area.

One account frequently invoked to explain findings such as these is that increased activation compensates for age-associated gray matter atrophy. We do not find strong support for this argument in examinations of ventral-lateral temporal-occipital cortex. Consider in this context the pattern of activation we observed in older adults with poorer task performance. Despite being matched in age, the older adults with better task performance demonstrated significantly greater ventral-lateral temporal-occipital activation during this semantic task compared to poorer-performing older adults. We used these functional data as a guide to examine relative atrophy in these age-matched groups, and we did not find significant differences in ventral-lateral temporal-occipital gray matter volume in these subgroups of older adults at the peak locus of activation (although there was a trend in this direction). An alternate possibility is that reduced frontal activation in poorer performing older adults resulted in underactivation of this occipital area, perhaps related to reduced white matter connectivity.

We observed increased ventral-lateral temporal-occipital activation for manufactured object compared to natural objects. Previous studies also have shown somewhat different activation patterns for manufactured and natural objects in this region (Chao and Martin, 2000). While the basis for this difference is the subject of some debate, we also found significantly greater activation for shape judgments than color judgments in a similar anatomic distribution. This is consistent with the importance of shape features in judgments of manufactured objects, and may reflect in part the greater activation of representations of shape features relative to color features during judgments of manufactured objects (Wierenga et al., 2009). We may not have seen a similar effect for color judgments because color may play a less distinctive role in judging a particular semantic category. Additional work will be needed to assess this issue. Regardless of the basis for these semantic category and perceptual feature effects, we did not observe any interaction of these effects with age within the group of older adults.

Finally, it is noteworthy that this study did not show activation in the anterior temporal lobe during performance of this measure of semantic memory (see also Bonner et al., 2013), although this must be interpreted cautiously due to the increased susceptibility artifact associated with BOLD imaging in the anterior temporal region (Devlin et al., 2000).

### Age-related activation changes in executive control regions during processing of object knowledge

There is increasing evidence supporting a role for executive processes in semantic memory (Grossman et al., 2002b; Jefferies et al., 2008; Reilly et al., 2011; Whitney et al., 2011a,b). We argued previously that several cognitive processes are likely to be recruited in young adults during semantic judgments that are involved in controlling the access and manipulation of stored representations of object knowledge (Grossman et al., 2013). Some have argued that these are domain-general executive functions, or processes within semantic memory that resemble these executive functions. Previous research thus has made a case for inferior frontal gyrus in the selection of items in the context of a semantic task (Thompson-Schill et al., 1997). We suggest that the role of dorsal portions of prefrontal cortex in semantic memory also includes functions related to acquiring new information and evaluating retrieved information about concepts in a rule-dependent manner (Grossman et al., 2002b; Koenig et al., 2005). These processes rely in part on regions that are also involved in domain-general strategic information processing beyond inferior frontal gyrus (Duncan, 2010; Woolgar et al., 2010, 2011).

In the present study, we observed activation in several domain-general executive regions during judgments of word meaning. This included dorsolateral prefrontal cortex, premotor cortex, and inferior parietal cortex. Moreover, we found that activation in several of these regions interacted with age and performance accuracy. Increased activation thus was seen in dorsal premotor, inferior frontal, and inferior parietal regions in the subset of older adults whose performance equaled that of young adults. Likewise, we also found greater frontal and inferior parietal activation in older adults with better performance compared to older adults with poorer performance. Since we did not experimentally manipulate executive processing demands, our conceptualization of the observed activity reflecting an aspect of modality-neutral executive control is based on the overall demands involved in our task. Our conclusions thus must be interpreted within this context. Nevertheless, this is similar to the pattern observed in ventral-lateral temporal-occipital regions associated with the representation of visual features of objects. This effect was not statistically robust in inferior frontal regions.

We hypothesize that the interaction of performance accuracy with domain-general activation provides a critical clue to the basis for age-associated differences in cognition. Specifically, we speculate that the process of selection associated with the inferior frontal region is less critical to successful semantic processing during healthy aging than increased activation in domain-general regions in dorsal premotor and inferior parietal cortices associated with the strategic way in which the information associated with a concept is evaluated. For example, premotor and inferior parietal activity may be related in part to short-term memory demands of keeping semantic information in mind while it is being evaluated.

Although increased activity may play a compensatory role in older adults' overall good performance on the semantic task, as commonly observed during many language and memory tasks in older adults (Cabeza et al., 2002; Wingfield and Grossman, 2006; Grady, 2008), the basis for this effect is unclear. Age-related activations often involve activations of contralateral, homologous brain regions, particularly in modality-specific tasks. For example, young adults may activate left prefrontal and parietal regions during performance of a verbal working memory task, but older adults are likely to activate prefrontal and parietal regions bilaterally (Reuter-Lorenz et al., 2000). Previous studies have implicated the contralateral homologue in the compensatory process, suggesting that increased recruitment of relevant brain tissue bilaterally allows task performance to be maintained despite increased age (Cabeza, 2002). The underlying assumption is that increases in activation compensate for age-related atrophy in relevant brain regions. The observations in the present study are largely consistent with this model. We identified a number of executive processing regions that suggested differences between better and worse performing older adults; of these, one (right precentral gyrus) showed significant gray matter atrophy in the worse performing older adults.

A hint about the mechanisms associated with age-related increased activation may be seen in previous work that examined language processing. In these studies, age-associated increases in bilateral activation were observed to some extent during language processing tasks such as sentence comprehension, although unilateral activation was seen during grammatical comprehension in young adults (Grossman et al., 2002a; Peelle et al., 2010). However, we have not observed this in all older adults. Thus, some older adults, including those with poorer sentence comprehension, showed unilateral activation of non-homologous regions, including non-language regions (Peelle et al., 2010; Tyler et al., 2010), particularly in prefrontal cortex (Grossman et al., 2002a). This raises the possibility that strategic control over processing may contribute to age-related changes in activation during language tasks such as semantic judgments.

In line with this, several investigators have suggested that increased activation in healthy aging may be related in part to support for a behavioral scaffolding process associated with improved performance (Cabeza et al., 2002; Wingfield and Grossman, 2006; Park and Reuter-Lorenz, 2009; Reuter-Lorenz and Park, 2010). The weak interaction between aging and judgments of semantic categories or perceptual features suggests that this hypothesized scaffolding process does not depend strongly on age-associated differences in the content of material being processed. Instead, scaffolding may depend more on the executive processes implemented to manipulate content. A similar effect also may be seen in the principles underlying “cognitive reserve.” This construct has been developed to explain different rates of change that can be seen in dementing conditions such as Alzheimer's disease (Stern, 2006). In some individuals, the rate of conversion from Mild Cognitive Impairment to Alzheimer's disease appears to be slowed, for example, and this is associated with increased education and superior job attainment. One argument is that these individuals have superior strategic processing that allows them to compensate by developing behavioral methods to optimize performance. Most relevant to the present study, patients with mild Alzheimer's disease show compensatory activity in executive regions for semantic memory tasks (Grady et al., 2003; Grossman et al., 2003; Wierenga et al., 2011). To the extent that strategic processing is related to domain general executive function, it may be that a similar form of cognitive reserve is operative in healthy aging that involves dorsal premotor activation during task performance in better-performing older adults.

## Conclusions

Our results lend support to the hypothesis that domain-general executive regions contribute critically to successful processing of semantic information during aging. This may reflect strategic processes involved in the retrieval, manipulation, and evaluation of representations of object concepts that are stored in modality-specific and heteromodal association cortices. Increased activation in frontal regions in some older adults may compensate in part for age-associated frontal atrophy. The observed prefrontal activity did not differ as a function of concept category or task, lending support to the hypothesis that this may reflect age-associated processing differences rather than changes in the representations of conceptual knowledge as we age.

### Conflict of interest statement

The authors declare that the research was conducted in the absence of any commercial or financial relationships that could be construed as a potential conflict of interest.
