# Supplementary material for: Age-related vulnerability in the neural systems supporting semantic processing
Source: Front Aging Neurosci. 2013 Sep 12;5:46. doi: 10.3389/fnagi.2013.00046 (PMC3770910; doi:10.3389/fnagi.2013.00046)
Supplement: Supplementary file 1 [file DataSheet1.PDF]

Supplemental Table 1. Maxima for fMRI activity for manufactured objects and natural kinds

Cluster size (μl)	Region	ROI Peak Coordinate X	Y	Z	Z score
**Manufactured objects**					
157080	Left occipital cortex	-42	-72	-12	> 8
	Left fusiform gyrus	-36	-82	-14	7.80
	Left occipital cortex	-42	-82	-4	7.76
26464	Left inferior frontal gyrus	-46	10	22	6.94
	Left precentral gyrus	-44	0	38	6.85
	Left inferior frontal gyrus	-46	6	30	6.82
3704	Brainstem	6	-28	-18	5.81
	Left thalamus	-24	-30	0	5.77
	Brainstem	-4	-26	-14	5.47
2592	Right thalamus	14	-12	6	5.79
	Right thalamus	14	-4	2	5.41
1528	Left thalamus	-12	-14	8	5.63
	Right thalamus	-20	-6	8	5.17
3512	Supplemental motor area	-2	16	48	5.50
	Supplemental motor area	-6	6	54	5.31
	Cingulate gyrus	8	12	46	5.23
264	Right middle frontal gyrus	36	-2	64	5.08
1080	Right insula	36	22	0	5.05
	Right insula	28	28	-6	4.93
	Right insula	28	26	6	4.60
576	Right precentral gyrus	40	2	28	5.03
**Natural kinds**					
16408	Left inferior parietal cortex	-28	-52	44	7.13
	Left inferior parietal cortex	-46	-38	46	6.15
	Left occipital cortex	-26	-66	30	6.05
136824	Left precentral gyrus	-44	8	32	7.06
	Left fusiform gyrus	-40	-60	-14	7.04
	Left precentral gyrus	-46	2	38	6.96
6904	Right precentral gyrus	42	4	30	6.18
	Right middle frontal gyrus	48	40	24	5.92
	Right inferior frontal gyrus	56	18	32	5.25
3752	Right insula	32	22	-2	6.09
	Right insula	28	22	10	5.15
2224	Right angular gyrus	32	-54	46	5.75
	Right inferior parietal cortex	40	-42	46	4.65
3744	Supplemental motor area	-4	16	48	5.60
	Cingulate gyrus	8	22	38	5.08
488	Cerebellum	0	-34	-3	5.39

Clusters from thresholding at voxelwise $p < .05$, FWE corrected, and an extent of 5 voxels

Supplemental Table 2. Maxima for fMRI activity comparing manufactured objects and natural kinds

Cluster size (μl)	Region	ROI Peak Coordinate			Z score
		X	Y	Z	
**Manufactured objects > natural kinds**					
8400	Left middle temporal gyrus	-52	-54	0	5.18
	Left inferior temporal gyrus	-50	-58	-8	4.77
	Left middle temporal gyrus	-52	-42	8	3.97
**Natural kinds > manufactured objects**					
3952	Left insula	-34	4	-14	5.28
	Left putamen	-28	-8	-4	4.22
	Left putamen	-32	-4	4	3.94

Supplemental Table 3. Maxima for fMRI activity for shape and color judgments

Cluster size (μl)	Region	ROI Peak Coordinate			Z score
		X	Y	Z	
**Shape**					
138168	Left fusiform gyrus	-44	-70	-16	> 8
	Left occipital cortex	-36	-84	-12	7.59
	Left occipital cortex	-48	-80	-6	7.36
25880	Left hippocampus	-36	-32	-10	6.85
	Left inferior frontal gyrus	-42	30	14	6.51
	Left inferior frontal gyrus	-42	10	22	5.86
2816	Basal ganglia	16	-4	4	5.71
	Right caudate	18	8	18	4.70
	Basal ganglia	20	-4	18	4.67
2056	Left thalamus	-12	-14	8	5.51
	Left basal ganglia	-16	2	4	4.46
2224	Right angular gyrus	32	-56	48	5.44
	Right middle occipital gyrus	32	-70	30	5.03
	Right middle occipital gyrus	32	-66	40	4.95
1248	Brainstem	0	-22	-16	5.44
1936	Right insula	32	22	-2	5.28
	Right insula	26	26	6	4.91
1216	Right inferior frontal gyrus	56	20	32	5.09
	Right inferior frontal gyrus	50	16	28	4.82
	Right inferior frontal gyrus	42	4	26	4.77
1240	Left lingual gyrus	-14	-32	-2	5.05
	Left hippocampus	-26	-28	-2	4.93
	Vermis	0	-44	2	4.58
488	Right middle frontal gyrus	48	40	24	4.93
	Right middle frontal gyrus	40	40	24	4.77
416	Left posterior middle temporal gyrus	-52	-42	8	4.87
	Left posterior middle temporal gyrus	-62	-36	6	4.65
48	Left anterior superior temporal gyrus	-54	12	-6	4.57
**Color**					
9992	Left inferior parietal cortex	-28	-52	44	6.97
	Left middle occipital gyrus	-26	-66	30	5.87
	Left inferior parietal cortex	-46	-36	46	5.12
51896	Left fusiform gyrus	-38	-62	-14	6.88
	Left fusiform gyrus	-30	-44	-20	6.59
	Left inferior occipital gyrus	-25	-94	-4	6.35
12256	Left precentral gyrus	-46	6	32	6.78
	Left inferior frontal gyrus	-44	28	18	5.40
	Left inferior frontal gyrus	-46	40	14	5.05

584	Left insula	-30	20	2	5.21
544	Right inferior frontal gyrus	42	6	28	5.20
536	Right angular gyrus	28	-52	42	5.10
600	Left hippocampus	-22	-26	-4	5.09
656	Supplemental motor area	-2	16	48	5.06
	Supplemental moto area	-8	6	54	5.06
344	Brainstem	6	-28	-18	5.04
	Brainstem	12	-16	-12	4.69
248	Right insula	30	22	4	4.97
264	Vermis	0	-58	-26	4.89
280	Brainstem	-6	-28	-16	4.85
80	Supplemental motor area	6	14	48	4.61

Clusters from thresholding at voxelwise $p < .05$, FWE corrected, and an extent of 5 voxels

Supplemental Table 4. Maxima for fMRI activity comparing shape and color judgments

Cluster size (μl)	Region	ROI Peak Coordinate			Z score
		X	Y	Z	
**Shape > color**					
14224	Left inferior occipital gyrus	-38	-86	-10	4.90
	Left fusiform gyrus	-40	-70	-12	4.50
	Left middle occipital gyrus	-44	-66	2	4.04
5200	Right inferior occipital gyrus	46	-82	-6	4.38
	Right fusiform gyrus	46	-62	-20	4.19
	Right fusiform gyrus	26	-70	-20	3.89
**Color > shape**					
(n/a)					
